# The Mediating Role of Resilience in the Relationship Between Anticipatory Grief and Quality of Life in Caregivers of People with Dementia: A Cross-Sectional Study

**DOI:** 10.3390/healthcare14010057

**Published:** 2025-12-25

**Authors:** Violeta Clement-Carbonell, Cristian A. Alcocer-Bruno, Nicolás Ruiz-Robledillo

**Affiliations:** Department of Health Psychology, University of Alicante, San Vicente del Raspeig, 03690 Alicante, Spain; violeta.clement@ua.es (V.C.-C.); nicolas.ruiz@ua.es (N.R.-R.)

**Keywords:** anticipatory grief, family caregivers, dementia, resilience, health-related quality of life, protective factors

## Abstract

**Background/Objectives:** Caring for dementia patients involves a significant emotional burden for family caregivers, who confront anticipatory grief (AG) processes that can negatively affect their health-related quality of life (HRQoL). This study examines the mediating role of resilience in the relationship between AG and HRQoL. **Methods:** A cross-sectional study was conducted with 144 family caregivers of people with dementia in the province of Alicante (Spain). Validated instruments were applied to measure AG (Caregiver Grief Scale), HRQoL (SF-12), and resilience (Brief Resilient Coping Scale). Descriptive analyses, Pearson correlations, and hierarchical regressions were used, as well as a mediation model based on Hayes’ PROCESS macro. **Results:** AG was negatively associated with resilience (*r* = −0.19, *p* = 0.025) and with both mental (*r* = −0.24, *p* = 0.004) and physical (*r* = −0.22, *p* = 0.009) components of HRQoL, whereas resilience was positively associated with mental HRQoL (*r* = 0.35, *p* < 0.001). In regression analyses, AG (B = −3.36, *p* = 0.006) and resilience (B = 1.16, *p* < 0.001) were significant predictors of mental HRQoL, explaining 30.4% of the variance (R^2^ = 0.30). Mediation analyses showed a significant indirect effect of AG on mental HRQoL through resilience (B = −1.28, 95% bootstrapped CI [−2.31, −0.84]), indicating partial mediation. **Conclusions:** Although AG negatively impacts HRQoL, resilience emerges as a relevant protective resource, especially for caregiver mental health. Therefore, it is crucial to promote resilient coping strategies in interventions that target this vulnerable population.

## 1. Introduction

Increased life expectancy has been linked to a higher risk of developing dementia, a highly prevalent illness that is expected to continue increasing over the coming years [1]. With the progressive aging of the population and the increased survival rate of people with chronic illnesses and disabilities, not only has the number of people requiring care increased, but this increased demand has also been accompanied by greater demands on its provision. This means that, over time, those with dementia require more support to meet their needs and perform their daily activities [2,3].

This support is usually provided by a caregiver who assists the sick person with their daily limitations [3]. In most cases, this caregiver is a family member (often a woman), such as a daughter or wife. The definition of the family caregiver (FC) includes any member of the family, friend, or partner who maintains a meaningful relationship with the patient and provides some form of care [4,5,6]. However, despite the impact of caring for a person with this condition, positive aspects associated with this task have also been described. These may improve the caregiver’s emotional well-being, coping skills, and satisfaction with the care [7,8]. Some of these aspects include the fact that, by attending to their family member, the caregiver spends time with them, strengthens their relationship and emotional bond, and may feel fulfilled by attending to and caring for them [9,10,11,12]. However, most studies have focused on the negative impact of caregiving on the health of the family member caring for the sick person. From a psychological point of view, the caregiver may experience depressive and anxiety symptoms, guilt, hopelessness, or anticipation and fear about the future [8,13]. From a physical point of view, caregivers may be at greater risk of suffering from sleep problems, inflammatory processes, and metabolic and cardiovascular diseases [7,14].

Given their high level of involvement in caregiving tasks and their close emotional bond with the patient, this population is vulnerable to high levels of distress during caregiving and bereavement [15,16,17,18]. In the case of dementia, family caregivers face complex situations. Most often, they have not received sufficient preparation to cope with these situations in an adaptive manner [19]. On the other hand, and given the progressive and irreversible deterioration that tends to accompany dementia, demands related to their care and attention increase. This may affect the caregiver’s health and well-being [20]. Caring for people with dementia has also been associated with negative physical, behavioral, and emotional consequences for the caregiver, including sleep problems [21,22,23,24,25]. These consequences may significantly affect the quality of life of these caregivers.

Family caregivers of people with dementia often experience a grieving process that begins at the time of diagnosis and evolves progressively as cognitive, relational, and functional losses accumulate. This phenomenon, known as anticipatory grief (AG)**,** refers to emotional, cognitive, and relational responses to ongoing and future losses associated with caregiving in the context of dementia [19,26,27,28]. Importantly, AG is conceptually distinct from pre-death grief, which encompasses emotional reactions emerging when death is imminent [26]. AG, by contrast, describes a broader and earlier process that includes changes in identity, role redefinition, and disruption of the caregiver–patient relationship across the trajectory of the illness. The progressive nature of dementia, characterized by impaired communication, increasing dependence, and the inability to resolve pending relational issues, makes AG particularly salient in this population [18,19,29]. Previous research has shown that AG, especially when combined with perceived losses and limited coping resources, may increase the risk of prolonged grief after death, with an estimated incidence of 10–15% [30].

Several studies have shown that AG can significantly affect caregivers’ physical and psychological health, including higher levels of depressive symptoms, anxiety, fatigue, sleep disturbances, and reduced perceived quality of life [28,29]. Recent research has highlighted that AG negatively influences health-related quality of life (HRQoL) by affecting well-being, autonomy, and subjective coping resources [31,32]. HRQoL is a multidimensional construct reflecting an individual’s perception of their physical and mental functioning in relation to their health status [33]. A substantial body of literature demonstrates that HRQoL in caregivers is shaped by both clinical and psychosocial factors, including multimorbidity, depressive symptoms, reduced functional autonomy, neurodegenerative disease in the care recipient, socioeconomic conditions, and personal adjustment variables [3,33,34]. Current research, however, focuses on aspects related to personal adjustment variables, such as protective factors for good physical and psychological health [35], with resilience being especially noteworthy [36,37,38,39].

Despite the negative effects of AG on caregivers’ well-being, the presence of protective psychological resources may mitigate its impact. One of the most consistently studied factors is resilience, defined as a dynamic process that enables individuals to adapt positively and recover psychological balance in the face of chronic stress and adversity [36,40]. In the context of dementia care, resilience has been shown to buffer stress responses, promote more adaptive cognitive appraisal of caregiving demands, and facilitate the use of functional coping strategies [37,39,40]. Higher levels of resilience in caregivers have been associated with greater perceived social support, higher self-efficacy, emotional stability, and lower levels of anxiety and depressive symptoms [36,41], positioning this construct as a key determinant of caregivers’ adjustment.

While prior research has examined AG, resilience, and HRQoL independently, no studies to date have simultaneously analyzed the mediating role of resilience in the relationship between AG and HRQoL in caregivers of people with dementia. Given the chronic and cumulative nature of loss in dementia, understanding how resilience may buffer the negative effects of AG is essential for developing targeted interventions and preventive strategies.

Therefore, the objective of this study is to analyze the mediating effect of resilience on the relationship between anticipatory grief and health-related quality of life in informal caregivers of individuals with dementia. We propose two hypotheses:

**H1.**  *Higher levels of AG will be associated with lower levels of HRQoL*.

**H2.**  *Higher levels of resilience will mediate the association between AG and HRQoL, buffering its negative impact*.

## 2. Materials and Methods

### 2.1. Study Design, Setting and Participants

This cross-sectional study was conducted in various associations for people with dementia in the province of Alicante. These associations operate at the county level within the province of Alicante and serve people with dementia and their families who live in that area. The diagnoses were made by neurology services specializing in dementia. The study included 144 family caregivers of people with dementia affiliated with these associations. The sample size was determined by the availability of eligible participants during the recruitment period. This sample size allows the detection of small-to-moderate associations and is appropriate for conducting regression-based mediation analyses using bootstrapping procedures, which are recommended for samples of this magnitude.

Inclusion criteria for participation in the study were (1) having a family member diagnosed with dementia; (2) identifying oneself as a family caregiver in a household with an individual suffering from dementia; (3) being over the age of 18; and (4) signing the informed consent form. Exclusion criteria included (1) sensory, physical, or mental deficits that hinder the participant’s understanding and completion of the assessment instruments; and (2) presence of psychiatric pathologies that may alter cognitive function and, therefore, influence performance on assessment instruments.

### 2.2. Procedure

At the start of the study, the research team contacted the associations of family members and people with dementia in the province of Alicante. Those who voluntarily decided to participate in the study were given detailed information on the research. In addition to completing and signing an informed consent form, they were assured the complete confidentiality of their data, as well as the anonymity of the participants through the assignment of a code to each of them.

After signing the informed consent, a psychologist specializing in health outcomes assessment administered the measurement instruments via tablet to the family caregivers participating in the study online, through the Google-Forms platform. Participants with limitations in the use of technology were given the questionnaire in paper format. The assessments were performed between January and May of 2024.

Inclusion criteria for participation in the study were (1) Having a family member diagnosed with dementia; (2) identifying oneself as a family caregiver in a household with an individual suffering from dementia; (3) being over the age of 18; and (4) signing the informed consent form. Exclusion criteria included (1) sensory, physical, or mental deficits that hinder the participant’s understanding and completion of the assessment instruments; and (2) presence of psychiatric pathologies that may alter cognitive function and, therefore, influence performance on assessment instruments.

This research study complies with the principles included in the Declaration of Helsinki (WMA, 2008 [42]). It has been carried out in accordance with the ethical requirements of research with human subjects (informed consent and right to information, personal data protection and confidentiality guarantees, no discrimination, free of charge, and the possibility of abandoning the study in any of its phases). The project received the authorization of the Ethics Committee of the University of Alicante prior to the start of its undertaking (UA-2023-02-03).

### 2.3. Variables and Measurements

#### 2.3.1. Sociodemographic and Clinical Characteristics

An ad hoc questionnaire was developed to collect descriptive information on caregivers and care recipients with dementia. The questionnaire assessed caregiver gender, age, marital status, educational level, and economic status, as well as care recipient gender, age, marital status, educational level, economic status, years since diagnosis, and illness stage. Age and years since diagnosis were recorded as continuous variables, whereas the remaining variables were collected using predefined categorical response options. The questionnaire was designed by the research team for sample characterization and was administered together with the standardized instruments included in the study.

#### 2.3.2. Anticipatory Grief in Caregivers of Individuals with Dementia

The Caregiver Grief Scale (CGS) was used to assess anticipatory grief in family caregivers of people with dementia [43]. It contains 11 Likert-like items with 5 categories ranging from 1 (totally disagree) to 5 (totally agree). The scale demonstrated good construct validity, supported by a robust four-factor structure confirmed through exploratory and confirmatory factor analyses, high internal consistency, strong test–retest reliability, and theoretically consistent associations with measures of depression, anxiety, and health-related quality of life. Moreover, the complete scale and its subscales demonstrated adequate levels of internal consistency (Cronbach’s alpha between 0.67 and 0.89). The scale includes four factors reflecting distinct aspects of caregiver grief: emotional pain (painful emotions related to the loss), relational loss (losses related to the relationship), absolute loss (anticipation of the future without the person), and acceptance of the loss (acceptance of the dementia and open expression of grief) [43]. CGS has no established cutoffs; higher scores indicate greater caregiver grief. Scores are typically interpreted relative to the sample or subscales to identify caregivers with low, moderate, or high grief. The version of the CGS used in this study was the Spanish forward–backward translation developed with a sample of caregivers of people with dementia in Spain [44]. In this study, the internal consistency index was α = 0.88.

#### 2.3.3. Health-Related Quality of Life

The Short Form Health Survey (SF-12) was used to assess HRQoL [45]. This instrument has been validated for the Spanish population and consists of 12 items derived from the 8 dimensions of the SF-36: general health, physical functioning, role limitations due to physical health, role limitations due to emotional problems, bodily pain, social functioning, mental health, and vitality. From these 8 dimensions, two summary components are obtained: Physical Health Component (PHC) and Mental Health Component (MHC). The items are coded on a scale ranging from 0 (worst state of health) to 100 (best state of health). This scale has shown good construct, convergent, and discriminant validity, with a well-established two-component structure (physical and mental health). The SF-12 scores are interpreted relative to population norms, with a mean of 50 and a standard deviation of 10; scores above 50 indicate better-than-average health, while scores below 50 suggest worse health. This approach allows identification of areas of physical or mental health vulnerability without relying on fixed clinical cutoffs. The SF-12 has proven to be a reliable and valid measure, having internal consistency estimates above 0.70 and significant correlations between versions [45]. Cronbach’s alpha of our study was 0.82, demonstrating adequate internal consistency.

#### 2.3.4. Resilience

A Spanish version of the Brief Resilient Coping Scale was used to assess the sample’s resilience level [46]. This scale was adapted for an elderly population [47]. The questionnaire consists of four items with a Likert-like scale of five points that assesses the adaptive ability of the subjects in the face of stressful situations (scale from 1 to 5, where 1 means the statement does not describe you at all and 5 means it describes you very well). This questionnaire has demonstrated solid psychometric validity. Its construct validity is supported by a clear unidimensional factorial structure, indicating that the scale captures a single, coherent dimension of resilient coping. Convergent validity has been established through positive associations with adaptive coping resources and indicators of psychological well-being. Taking into account the total score, individuals can be classified into low resilience (score of 13 or less), medium resilience (score between 14 and 16), and high resilience (score of 17 or more). The Spanish version has demonstrated an adequate reliability and validity, with a Cronbach’s alpha of 0.86 [47]. In this study, it reached 0.81.

### 2.4. Data Analysis

Descriptive analyses of sociodemographic and clinical characteristics were performed on both the caregiver sample and the sample of dementia sufferers. Pearson correlations were used to analyze the relationship between the variables under study. Assumptions of linearity and absence of extreme outliers were examined prior to correlation analyses. Hierarchical regression analyses were also carried out. Assumptions for linear regression were examined prior to hypothesis testing. Independence of residuals was confirmed using the Durbin–Watson statistic (values close to 2). Multicollinearity was not a concern, as tolerance values exceeded 0.40 and variance inflation factors were below 2.5. Visual inspection of histograms and normal probability plots indicated approximate normality of residuals. Linearity and homoscedasticity were supported by scatterplots of standardized residuals versus predicted values, which showed no systematic patterns. Finally, no influential observations were detected, as Cook’s distance values were well below recommended thresholds. For the analysis of mediation, the model 4 of Hayes’ PROCESS macro was used [48]. This macro is a trajectory model analysis tool used to estimate direct and indirect effects in mediation models. It is an empirical, bias-corrected bootstrapping procedure that approximates confidence intervals based on the repeated resampling of observed data. A measurement effect is only significant when the 95% confidence interval does not include zero. In this case, it is found that in 95% of the bootstrapped samples, the effect of AG on HRQoL is mediated by the resilience effect. In this study, resampling was performed 10,000 times, as recommended by Hayes [48]. In small samples, bootstrapping has been shown to be the most effective and powerful method for testing indirect effects, as compared to other traditional methods, such as linear regression or the Sobel test. In all of the cases, *p* < 0.05 was considered significant. All of the statistical analyses were carried out with SPSS, version 24.0 (IBM Corp., Armonk, NY, USA) [49].

### 2.5. Bias

Several strategies were implemented to reduce potential sources of bias. Standardized and validated instruments were used to minimize measurement bias, and data collection followed a uniform protocol across all participants. Although the cross-sectional design precludes causal inferences and self-reported measures may be subject to recall or social desirability bias, these limitations are common in caregiver research and were addressed by including relevant covariates in the analyses. Additionally, recruitment through dementia associations may limit generalizability; however, this approach ensured access to a well-characterized and clinically relevant caregiver population.

## 3. Results

### 3.1. Sociodemographic and Clinical Characteristics of the Sample

Table 1 summarizes the descriptive statistics (means, proportions, and standard deviations) of the sociodemographic and clinical characteristics of all of the participants, including both family caregivers and people with dementia. The final sample consisted of 144 subjects, 38 men (26.4%) and 106 women (73.6%). The average age of the participants was 59.70 (SD = 11.41) and 80.16 for the individuals with dementia (SD = 9.29). The marital status of the majority of the family caregiver sample was married (69.4%, *n* = 100), as was the case with the individuals with dementia (50%, *n* = 72). Regarding education level, a significant percentage of the participants had primary or lower levels of education (40.3%), followed by secondary school and high school studies (33.4%), and university-level studies (26.4%). In the case of people with dementia, most had primary education or less (71.5%). The income level of most participants was between 1000 and 2000 euros, compared to people with dementia, who do not exceed 1500 euros per month. Regarding the stage of the illness, most people with dementia were in a moderate stage (59.7%, *n* = 86).

### 3.2. Initial Analysis of Correlations Between Anticipatory Grief, HRQoL, and Resilience

Before conducting the mediation analyses, descriptive statistics and bivariate correlations between the main study variables were examined (Table 2). All scales demonstrated satisfactory internal consistency, with Cronbach’s alpha coefficients above 0.80, indicating robust reliability. Descriptive statistics revealed mean scores and standard deviations within a moderate range for all constructs assessed.

Bivariate correlation analyses showed that anticipatory grief was negatively associated with resilience (*r* = −0.19, *p* = 0.025) and with both the physical (*r* = −0.21, *p* = 0.009) and mental (*r* = −0.23, *p* = 0.004) components of health-related quality of life. In contrast, resilience was positively associated with mental HRQoL (*r* = 0.35, *p* < 0.001) (Table 2).

### 3.3. Multiple Regression Analysis Between Anticipatory Grief and Health-Related Quality of Life

A hierarchical regression analysis was performed to examine the extent to which sociodemographic variables (age, gender, marital status, educational level, employment status, income, and illness stage) predicted the physical component of quality of life (PCS_SP) (Table 3). The sociodemographic variables introduced in Step 1 explained 22.7% of the variance (F(7,136) = 5.70, *p* < 0.001). The inclusion of AG (CGS_TOTAL) in Step 2 resulted in a slight, though not significant, improvement of the model (ΔR^2^ = 0.020, ΔF(1,135) = 3.65, *p* = 0.058). Upon adding resilience (BRCS_TOTAL) in Step 3, the change remained insignificant (ΔR^2^ = 0.005, ΔF(1,134) = 0.84, *p* = 0.362). In this final model, education level (β = 0.297, *p* = 0.001) and marital status (β = −0.185, *p* = 0.026) were the only significant predictors. Neither AG (β = −0.138, *p* = 0.087) nor resilience (β = 0.07, *p* = 0.362) were significant predictors of the physical component. The final regression model showed a significant overall fit (F(9,134) = 5.01, *p* < 0.001) and explained 25.2% of the variance in the physical component of health-related quality of life (adjusted R^2^ = 0.202).

A second hierarchical regression analysis was performed to identify predictors of the mental component of health (MCS_SP) (Table 4). In the first step, sociodemographic covariates (age, gender, marital status, educational level, employment status, income, and illness stage) were introduced, explaining 16.0% of the variance in mental health (F(7,136) = 3.71, *p* = 0.001). In the second step, AG was added (CGS_TOTAL), significantly improving the explained variance (ΔR^2^ = 0.063, ΔF(1,135) = 11.03, *p* = 0.001). In the third step, resilience was added (BRCS_TOTAL), which produced a new significant increase in the explained variance (ΔR^2^ = 0.081, ΔF(1,134) = 15.55, *p* < 0.001). The final model explained 30.4% of the total variance in the mental health component of quality of life (adjusted R^2^ = 0.258). In this final model, both AG (β = −0.213, *p* = 0.006) as well as resilience (β = 0.303, *p* < 0.001) were significant predictors. Higher levels of AG were associated with worse mental health, while greater resilience was linked to better psychological functioning. Of the sociodemographic variables, marital status (β = 0.121, *p* = 0.038) and stage of the illness (β = −0.194, *p* = 0.009) were significant. The final regression model showed a significant overall fit (F(9,134) = 6.52, *p* < 0.001) and explained 30.4% of the variance in the mental component of health-related quality of life (adjusted R^2^ = 0.258).

### 3.4. Mediation Analyses

A simple mediation model (Model 4 in PROCESS for SPSS, version 3.5.3; Hayes, 2017 [48]) was estimated to examine whether resilience mediated the relationship between AG and the mental component of quality of life (Table 5). Age, sex, marital status, education level, employment status, income, and stage of the illness were entered as covariates in the model. The analysis used 10,000 bootstrap samples with 95% percentile confidence intervals.

The regression model predicting resilience was significant, F(8,135) = 2.29, *p* = 0.025, explaining 11.9% of the variance. AG negatively predicted resilience (β = –0.70, SE = 0.35, t = –2.00, *p* = 0.048, 95% CI [–1.38, –0.01]), indicating that higher anticipated grief was associated with lower resilience. Among the covariates, only marital status was significant (β = 0.77, *p* = 0.002) in this regression model. The model predicting the mental health component of HRQoL was significant, F(9,134) = 6.52, *p* < 0.001, accounting for 30.4% of the variance (R^2^ = 0.30). Both AG (β = –3.36, SE = 1.21, t = –2.78, *p* = 0.006, 95% CI [–5.75, –0.97]) and resilience (β = 1.16, SE = 0.29, t = 3.94, *p* < 0.001, 95% CI [0.58, 1.74]) significantly predicted mental health. Thus, greater resilience was related to better mental health, whereas higher AG was associated with poorer mental health. Regarding covariates, education level (β = –1.05, *p* = 0.038) and stage of the illness (β = –4.15, *p* = 0.009) showed significant effects on the mental health component of HRQoL.

**Figure 1:**
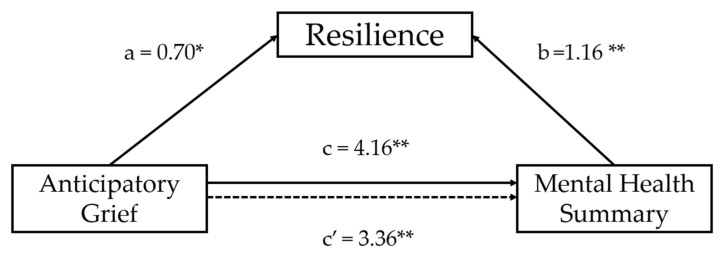
Results of multiple mediation analyses exploring the mediating effect of on the association between anticipatory grief and Mental Health Component controlling for age, sex, marital status, education level, employment status, income, and stage of the illness as covariates (** *p* < 0.01; * *p* < 0.05). The presented values are standardised regression coefficients where “a” indicates the standardised regression coefficient in the association between anticipatory grief and resilience; “b” indicates the standardised regression coefficient in the association between resilience and Mental Health Component; “c” indicates an indirect effect and “c′” represents a direct effect in the association between anticipatory grief and Mental Health Component. Dashed line represents non-significant effects.

The total effect of AG on mental health was significant (B = –4.16, SE = 1.25, t = –3.32, *p* = 0.001, 95% CI [–6.64, –1.68]), explaining 22.4% of the variance. When resilience was included, the direct effect of AG remained significant (B = –3.36, *p* = 0.006), and the indirect effect of anticipatory grief on the mental component of health-related quality of life through resilience was significant (B = −1.28, BootSE = 0.42, 95% bootstrapped CI [−2.31, −0.84]), indicating a partial mediation. The completely standardized indirect effect was β = −0.05 (95% BootCI [−0.12, −0.00]), supporting the mediating role of resilience in the association between AG and mental health (Figure 1).

## 4. Discussion

This study aimed to analyze the mediating role of resilience in the relationship between AG and HRQoL in family caregivers of people with dementia. The results confirm our main hypotheses: higher levels of AG were associated with lower perceived resilience and worse HRQoL, especially in its mental dimension. It was also found that resilience partially mediates this relationship, highlighting its relevance as a psychological resource linked to lower levels of psychological distress in the caregiving context.

Regarding effect size interpretation, the associations observed in this study ranged from small to moderate in magnitude. Anticipatory grief showed small-to-moderate negative effects on mental health–related quality of life, whereas resilience exhibited a moderate protective effect. Importantly, the final regression model explained approximately 30% of the variance in mental HRQoL, which represents a substantial explanatory capacity within psychosocial research contexts involving complex caregiving processes.

In line with previous research, our results corroborate the idea that AG is a significant and multidimensional emotional experience, closely related to the functional and relational decline of the family member with dementia [19,43,50]. Specifically, the emotional pain subscale (CGS_EP), which captures the affective experience of progressive loss, revealed an especially strong negative association with mental quality of life, reinforcing its specific weight in the deterioration of the caregiver’s psychological well-being. This result is consistent with recent studies that have identified emotional pain as a key dimension of AG. It has been shown to have adverse effects on mental health, coping strategies, and the perception of social support [28,31].

In addition to emotional pain, other dimensions of AG also have significant associations with health variables. For example, the absolute loss subscale (CGS_AL), which reflects the expectation of a future without the person being cared for, is negatively associated with physical and mental quality of life. This suggests that the cognitive anticipation of loss is linked to caregivers’ physical and psychological well-being. These findings support other studies, such as those carried out by Nielsen et al. (2016) [51], which suggested that a persistent sense of loss and future disconnection is associated with nonspecific physical symptoms, such as insomnia and body tension, even prior to the death of a loved one.

The acceptance of loss subscale (CGS_AOL) was also negatively associated with physical and mental quality of life. This indicates that lower levels of acceptance may be related to poorer emotional and somatic adjustment to the care process. This pattern aligns with recent research, suggesting that a lack of acceptance in the context of geriatric care may act as a barrier to adaptive coping and is associated with greater somatization and emotional distress [52,53,54].

It is noteworthy that these correlations remain significant even when controlling for resilience, which reinforces the robustness of the link between the different components of AG and the caregiver’s quality of life. The overall component of AG (CGS_TOTAL) had negative correlations with both the physical and the mental dimensions of HRQoL. This reinforces the accumulated evidence on the transversal effect of AG on the psychosocial well-being of the caregiver [8,55].

This multidimensional pattern of relationships has also been documented in systematic reviews, highlighting the idea that caregivers of people with dementia who have high levels of AG experience a greater perceived burden, functional impairment, social isolation, and a greater likelihood of developing affective and somatic disorders [14,30,56]. Thus, the empirical evidence obtained in this study reinforces the clinical value of assessing not only global AG, but also its different dimensions, in order to intervene more specifically and effectively in the process of supporting family caregivers.

Regarding the role of resilience, the results of this study provide relevant empirical evidence on its protective function in the context of informal care for people with dementia. First, a moderate positive correlation was observed between resilience and mental well-being, indicating that caregivers who perceive higher levels of resilience experience greater psychological well-being. This finding is consistent with numerous studies that have described resilience as an essential psychological resource that promotes effective coping with the chronic stress resulting from long-term caregiving [55,57].

The results also provide relevant information on the role of sociodemographic covariates. In the mental component of quality of life model (MCS_SP), marital status and illness stage were significant predictors. Married caregivers or those in relationships demonstrated greater levels of psychological well-being. This suggests that emotional and relational support play a protective role against the impact of AG. This finding coincides with the literature that identifies social support as a mitigating factor against caregiver stress and a predictor of improved mental health [58,59,60]. Conversely, being in more advanced stages of the illness was associated with worse mental health, possibly due to an increased care burden, the functional decline of the family member, and the accumulation of symbolic losses [61].

As for the physical component of quality of life (PCS_SP), level of education was the main positive predictor, suggesting that caregivers with higher levels of education reported better physical quality of life. This may be explained by the relationship between education and the use of health resources, health literacy, and the adoption of preventive strategies [62]. In turn, caregivers without a partner (single, widowed, or separated) displayed worse physical functioning, possibly linked to a lower availability of instrumental support in caregiving tasks [63]. These associations reinforce the need to consider the structural and relational conditions of the caregiver in the assessment of their well-being.

On the other hand, variables such as age, gender, employment status, and income did not display significant effects upon controlling for psychosocial variables. This suggests that their influence is indirect or mediated by other factors, such as resilience or social support. This finding is consistent with studies that have indicated that personal psychological resources explain more of the variability in caregivers’ mental health than traditional sociodemographic factors [36,37,39,64].

The mediation analysis performed using the bootstrap model confirmed that resilience partially mediates the relationship between AG (CGS_TOTAL) and mental quality of life. The indirect effect was significant, while the direct effect, although reduced, remained significant, indicating partial mediation. These findings are in line with recent research that has proposed similar mediation models. In these models, resilience acts as a buffer against the psychological impact of grief, burden, or relational uncertainty on caregivers [53,56].

It should be noted that, in our study, resilience did not show significant correlations with physical quality of life, suggesting that its influence is mainly concentrated in the emotional and cognitive domains of well-being. This dissociation has also been reported in other studies [21,22], which have argued that the physical consequences of caregiving (e.g., fatigue, musculoskeletal pain, sleep disorders) are more dependent on structural factors (such as the duration of care, the availability of formal support, or the functional condition of the patient) than on the psychological resources of the caregiver.

Furthermore, significant negative correlations were found between resilience and various dimensions of AG, especially with the emotional pain subscale and the absolute loss subscale. This suggests that more resilient caregivers tend to experience lower levels of anticipatory emotional distress and have less difficulty envisioning a future without their loved one. These results are consistent with studies that have identified resilience as a negative predictor of anticipatory anxiety and a moderating factor with respect to the risk of complicated grief [35,51].

Caregivers with high resilience tend to display a greater capacity to adapt to progressive loss, as well as a greater ability to use more functional coping strategies (e.g., cognitive reappraisal, seeking support) and to preserve their social network. This results in less anxiety and depressive symptoms and a lower perception of burden [36,65]. These elements explain why resilience acts as an effective mediator in protecting psychological well-being, although not on a physical level.

Finally, our results suggest that promoting resilience is not only desirable but also clinically useful. Existing interventions targeting dementia-related grief commonly combine psychoeducation with cognitive–emotional strategies from CBT, acceptance-based approaches, and mindfulness [66]. These programs yield small-to-moderate but significant reductions in grief and also improve caregiver burden, depression, empowerment, and resilience, suggesting that addressing anticipatory or pre-death grief may produce broader benefits for caregivers’ psychological well-being [44,67]. Interventions intended to strengthen personal resources such as self-efficacy, emotional acceptance, and cognitive flexibility may have an indirect positive impact on the mental health of caregivers, mitigating the negative effects of AG and fostering healthier means of coping.

However, resilience did not demonstrate a significant mediating effect on the physical dimension of HRQoL. This lack of effect may be due to the fact that physical symptoms associated with prolonged caregiving, such as fatigue, musculoskeletal problems, or insomnia, are more closely linked to structural factors (e.g., the intensity of care, the length of the workday, the lack of external support) than to individual psychological resources [21,22]. This finding highlights the need to intervene not only with respect to the personal variables, but also on the contextual conditions making up the caregiving experience.

From a practical perspective, these findings have important implications for both caregivers and the professionals who work with them. The identification of anticipatory grief and resilience as key variables associated with caregivers’ quality of life underscores the need for early detection strategies aimed at identifying caregivers at risk of elevated AG, particularly in its emotional pain and absolute loss dimensions. Healthcare and social care professionals could benefit from incorporating routine screening for AG into clinical and community settings, allowing for timely referral to targeted support services. In parallel, intervention programs specifically designed to strengthen resilience, such as resilience training workshops, emotional regulation interventions, structured psychoeducational programs, and support groups focused on anticipatory grief, may help caregivers develop adaptive coping strategies and reduce psychological distress. Emotional support interventions, including individual or group-based counseling and peer-support initiatives, as well as psychoeducational actions aimed at increasing awareness and understanding of AG as a normative process in dementia caregiving, may further contribute to improving caregivers’ psychological well-being. Together, these approaches illustrate how the present findings can be translated into concrete actions with the potential to produce meaningful improvements in caregivers’ quality of life.

In conclusion, the findings of this study reinforce the need to implement psychoeducational programs for family caregivers that address both the recognition and validation of AG and the strengthening of resilient coping resources. Another priority should involve promoting more sustainable and sensitive care environments that address the emotional distress of caregivers. This may include formal support services, mutual support groups, and community-based support strategies. Understanding AG allows clinicians and researchers to better identify caregiver needs, develop targeted interventions, and support families throughout the entire trajectory of dementia, not just at the end of life.

Among the main limitations of the present study, it is important to note, first, a potential selection bias, as the sample was recruited exclusively through associations of relatives and caregivers of people with dementia. This may imply an overrepresentation of caregivers who are more engaged, have greater access to formal resources, or present specific levels of social support, thereby limiting the generalizability of the findings to the broader population of informal caregivers. Second, the use of two modes of administration of the assessment instruments (digital format via tablet and paper-based format) may have introduced mode effects, particularly in subjective variables, given that differences in digital literacy, comprehension, or fatigue could have influenced participants’ responses. In addition, the study did not include several relevant covariates that have been identified in the literature as potentially influential in anticipatory grief, resilience, and caregivers’ quality of life, such as daily hours of care, type of dementia, level of objective caregiver burden, presence of depressive symptoms, perceived social support, or the relationship between the caregiver and the care recipient. The absence of these variables limits the ability to control for potential confounding factors and to more precisely understand the mechanisms underlying the observed associations.

Future studies may wish to delve deeper into the differential mechanisms of AG on the physical and mental dimensions of quality of life. They may also incorporate standardized measures of somatic symptomatology and utilize longitudinal designs to explore the evolution of AG throughout the process of the illness.

## 5. Conclusions

The results of this study confirm that AG is a complex emotional phenomenon that significantly affects the quality of life of family caregivers of people with dementia, especially in its psychological dimension. Among the various dimensions evaluated, emotional pain stands out as a central component of AG. This pain has been strongly linked to a decline in mental well-being. Furthermore, resilience was observed to exert a partial moderating effect in this relationship, acting as a key psychological resource that mitigates the adverse effects of AG on mental health. Taken together, these findings underscore the relevance of AG and resilience as central mechanisms underlying caregivers’ psychological adjustment, highlighting their joint contribution to explaining variations in mental quality of life in this population.

In contrast, no mediating effect of resilience was found in the relationship between geriatric assessment and physical quality of life. This suggests that the somatic consequences of prolonged caregiving may depend on more structural or contextual factors, such as the intensity of care, the availability of formal support, or the functional status of the family member. These findings highlight the need to address the emotional and physical aspects of caregiving in an integrated manner. It is necessary to incorporate intervention strategies that are aimed at improving emotional acceptance, strengthening resilience, and alleviating the physical and organizational burdens of caregiving. Overall, the present results provide clinically meaningful evidence that can inform the development of more comprehensive and targeted interventions aimed at improving caregivers’ overall quality of life.

## 6. Future Lines of Research

Given the cross-sectional nature of this study, future studies may wish to adopt longitudinal designs permitting the observation of the evolution of AG throughout the course of the illness and after the death of the family member. These longitudinal studies may identify factors that predict more or less healthy adaptation trajectories.

It may also be useful to include clinical measures of somatization or biomarkers of physiological stress (e.g., cortisol, blood pressure, inflammatory markers) to more precisely examine the somatic pathway of general acceptance and its relationship with emotional acceptance. Another recommendation involves examining the mediating or moderating role of other relevant psychological variables, such as self-efficacy, sense of coherence, dispositional optimism, or perceived social support.

Finally, it is necessary to validate and implement psychoeducational interventions aimed at fostering resilience, emotional regulation, and the processing of general grief, in order to improve the overall well-being of caregivers and reduce the risk of complicated grief following loss.

## Figures and Tables

**Table 1:** Mean and standard deviation, and frequency and percentage in sociodemographic and clinical characteristics of the sample.

Variable/Characteristics		*n* = 144	% Sample
**Caregiver**			
Gender	Male	38	26.4
	Female	106	73.6
Age	Mean	59.70	
	SD	11.413	
Marital status	Single	25	17.4
	Married	100	69.4
	Divorced	13	9
	Widowed	6	4.2
Education level	Read and write	14	9.7
	Primary school	44	30.6
	Secondary school	25	17.4
	High school	23	16
	University and above	38	26.4
Economic status	<1000 euros	23	16
	1000–1500 euros	41	28.5
	1501–2000 euros	31	21.5
	2001–2500 euros	21	14.6
	>2500 euros	28	19.4
**Person with dementia**			
Gender	Male	42	29.2
	Female	102	73.6
Age	Mean	80.16	
	SD	9.287	
Marital status	Single	5	3.5
	Married	72	50
	Divorced	4	2.8
	Widowed	63	43.8
Academic status	Read and write	71	49.3
	Primary school	32	22.2
	Secondary school	14	9.7
	High school	15	10.4
	College and above	12	8.3
Economic status	>1000 euros	45	31.3
	1000–1500 euros	39	27.1
	1501–2000 euros	28	19.4
	2001–2500 euros	13	9
	+2500 euros	19	13.2
Years since diagnosis	Mean	5.23	
	SD	4.19	
Illness stage	Low	21	14.6
	Moderate	86	59.7
	High	37	25.7

**Table 2:** Descriptive statistics and Pearson’s *r* correlations between the investigated variables.

	M (SD)	Range	α	2	3	4	5	6	7	8
1. BRCS_TOTAL	13.50 (3.5)	4–20	0.81	−0.18 *	−0.09	−0.18 *	−0.14	−0.19 *	0.35 **	0.06
2. CGS EP	3.36 (0.96)	1–5	0.67	-	0.54 **	0.41 **	0.52 **	0.77 **	−0.39 **	−0.27 **
3. CGS RL	3.87 (1.12)	1–5	0.89		-	0.48 **	0.48 **	0.81 **	−0.20 *	−0.14
4. CGS AL	3.01 (1.1)	1–5	0.83			-	0.61 **	0.80 **	−0.25 **	−0.26 **
5. CGS AOL	3.39 (1.12)	1–5	0.62				-	−0.79	−0.31 **	−0.35 **
6. Total Anticipatory Grief	3.41 (0.85)	1–5	0.88					-	−0.24 **	−0.22 **
7. SF-12 Mental Health	17.10 (4.08)	3.06–69.56	0.75						-	0.47 **
8. SF-12 Physical Health	14.60 (3.08)	6.77–70.15	0.80							-

Note: BRCS_TOTAL: resilience; CGS EP: Emotional pain; CGS RL: relational loss; CGS AL: absolute loss; CGS AOL: acceptance of loss; SF-12 Mental Health: mental health component; SF-12 Physical Health: physical health component. (** *p* < 0.01; * *p* < 0.05).

**Table 3:** Hierarchical regression analysis for predicting physical quality of life (PCS_SP).

Model	Variable	B	Standard Error	β	t	*p*
1	Constant	57.54	8.16	—	7.05	<0.001
	Age	−0.20	0.11	−0.19	−1.74	0.085
	Sex	0.25	2.16	0.009	0.11	0.909
	Marital status	−1.43	0.73	−0.156	−1.96	0.052
	Education level	1.55	0.45	0.309	3.44	0.001
	Employment status	−0.34	0.44	−0.087	−0.76	0.449
	Income	−0.13	0.75	−0.016	−0.18	0.860
	Stage of illness	−0.57	1.39	−0.031	−0.41	0.683
2	Constant	63.15	8.59	—	7.35	<0.001
	Age	−0.21	0.11	−0.21	−1.86	0.065
	Sex	0.67	2.15	0.03	0.31	0.754
	Marital status	−1.51	0.73	−0.16	−2.08	0.039
	Education level	1.49	0.45	0.30	3.34	0.001
	Employment status	−0.17	0.45	−0.04	−0.37	0.708
	Income	−0.18	0.75	−0.02	−0.24	0.807
	Stage of illness	−0.11	1.40	−0.01	−0.08	0.939
	CGS_TOTAL	−2.03	1.06	−0.15	−1.91	0.058
3	Constant	60.25	9.16	—	6.57	0.000
	Age	−0.22	0.11	−0.22	−1.93	0.055
	Sex	0.69	2.15	0.03	0.32	0.748
	Marital status	−1.70	0.75	−0.18	−2.25	0.026
	Education level	1.49	0.45	0.30	3.32	0.001
	Employment status	−0.13	0.45	−0.03	−0.28	0.781
	Income	−0.20	0.75	−0.02	−0.27	0.785
	Stage of illness	−0.15	1.40	−001	−0.11	0.916
	CGS_TOTAL	−1.86	1.08	−0.14	−1.73	0.087
	BRCS_TOTAL	0.24	0.26	0.07	0.92	0.362

Note: CGS_TOTAL: Total anticipatory grief; BRCS_TOTAL: total resilience.

**Table 4:** Hierarchical regression analysis for predicting mental quality of life (MCS_SP).

Model	Variable	B	Standard Error	β	t	*p*
1	Constant	33.71	9.88	—	3.41	0.001
	Age	0.15	0.14	0.13	1.10	0.272
	Sex	−1.23	2.61	−0.04	−0.47	0.638
	Marital status	2.35	0.89	0.22	2.65	0.009
	Education level	−0.89	0.55	−0.15	−1.64	0.103
	Employment status	0.27	0.54	0.06	0.51	0.613
	Income	0.70	0.91	0.07	0.77	0.443
	Stage of illness	−4.91	1.69	−0.23	−2.91	0.004
2	Constant	45.22	10.14	—	4.46	<0.001
	Age	0.13	0.13	0.11	0.95	0.345
	Sex	−0.35	2.54	−0.12	−0.14	0.889
	Marital status	2.18	0.86	0.20	2.55	0.012
	Education level	−1.01	0.53	−0.17	−1.91	0.058
	Employment status	0.62	0.53	0.14	1.17	0.244
	Income	0.60	0.88	0.06	0.68	0.496
	Stage of illness	−3.96	1.65	−0.18	−2.39	0.018
	CGS_TOTAL	−4.16	1.25	−0.26	−3.32	0.009
3	Constant	31.23	10.27	—	3.04	0.003
	Age	0.08	0.13	0.70	0.64	0.524
	Sex	−0.27	2.41	−0.01	−0.11	0.912
	Marital status	1.29	0.85	0.12	1.53	0.128
	Education level	−1.05	0.52	−0.18	−2.10	0.038
	Employment status	0.82	0.51	0.18	1.63	0.105
	Income	0.49	0.84	0.05	0.59	0.556
	Stage of illness	−4.15	1.57	−0.19	−2.64	0.009
	CGS_TOTAL	−3.36	1.21	−0.21	−2.78	0.006
	BRCS_TOTAL	1.16	0.29	0.30	3.94	<0.001

Note. CGS_TOTAL: Total anticipatory grief; BRCS_TOTAL: total resilience.

**Table 5:** Mediation analysis: Indirect effect of AG (CGS_TOTAL) on Mental Health Component (CS_SP) through resilience (BRCS_TOTAL).

Effect	B	Standard Error	LLCI	ULCI	*p*
Total effect (c)	−4.16	1.25	−6.63	−1.69	0.004
Direct effect (c′)	−3.36	1.21	−5.75	−0.97	0.006
Indirect effect (a × b)	−1.28	0.42	−2.31	−0.84	—

Note: A bootstrap procedure with 10,000 samples was used. LLCI = Lower limit of the confidence interval; ULCI = Upper limit of the confidence interval.

## Data Availability

The data presented in this study are available on request from the corresponding author due to containing sensitive personal information about the participants.
